# Intensity of adoption of integrated pest management practices in Rwanda: A fractional logit approach

**DOI:** 10.1016/j.heliyon.2022.e08735

**Published:** 2022-01-10

**Authors:** Vincent Gadamba Misango, Jonathan Makau Nzuma, Patrick Irungu, Menale Kassie

**Affiliations:** a Department of Agricultural Economics, University of Nairobi, Kenya; b International Centre of Insect Physiology and Ecology (*icipe*), Kenya

**Keywords:** Fall armyworm, Fractional logit model, Intensity of adoption, Push-pull technology, Stemborer

## Abstract

The push-pull technology (PPT) is considered as an alternative integrated pest management strategy for the control of fall armyworm and stemborer, among smallholder maize farmers in sub-Sahara African to conventional pesticides. However, the extent of PPT use in Rwanda where the technology was introduced in 2017 remains largely unexplored. This paper employed a fractional logit model to assess the factors influencing the intensity of adoption of PPT among smallholder maize farmers in Gatsibo and Nyagatare districts of Rwanda using survey data obtained from 194 PPT adopter households selected using a cluster sampling technique. While only 5 percent of smallholder farmers in Rwanda have adopted PPT as an integrated pest management strategy, on the average, these farmers cultivated 26 percent of their maize plots to the technology. Our results show that the perceived benefits of PPT, its perceived effectiveness in pest control, group membership, livestock ownership, and gender of the farmer had significant effects on the intensity of adoption of the PPT in Rwanda. These findings give compelling evidence to recommend that development initiatives should give emphasis on creating awareness on the perceived benefits of PPT adoption using group approaches that are gender disaggregated.

## 1 Introduction

Low agricultural productivity emanating from both biotic and abiotic constraints remains a key challenge for smallholder rural farmers in the sub-Sahara African (SSA) (Murage et al., 2015a; Midega et al., 2015). Abiotic constraints such as droughts, unpredictable weather patterns, climate change and limited access to quality inputs (seeds, fertilizer and chemicals) have continuously limited agricultural productivity in the region (AGRA, 2014). Biotic constraints (living organisms that shape the ecosystem and comprise of soil organisms) include on the one hand pest (both storage and field) and disease incidents such as the maize lethal necrotic disease (MLND) and predators such as mites, moles, locust, birds etc. and on the other hand, field pest specifically the fall armyworm and stemborer pests (AGRA, 2014; Midega et al., 2015). The low productivity among smallholder maize farmers in SSA is exacerbated by high post-harvest losses that are estimated at up to 24 percent of output without any intervention (Affognon et al., 2015).

Fall armyworm (FAW) and stemborer pests remain the most important field pests in Eastern Africa owing to their negative economic impacts on maize production, the main food staple in the Eastern African region (Midega et al., 2015; Kumela et al., 2018; IITA, 2019). The FAW moth (*Spodoptera frugiperda*) originated from the tropics and sub-tropics of America in early 2016 and spread to West and Central Africa in late 2016 and even later to other parts of Eastern, Northern, and Southern Africa (Goergen et al., 2016; Midega et al., 2018). The moth lays eggs that hatch into larvae that eat leaves at night and hide in the maize funnel during the day (Day et al., 2017). Stemborer (*Chilo partellus*) is a native of Asia that spread into Eastern, Southern, and Central Africa in the early 1930s and is now endemic in SSA (Harris, 1990; Midega et al., 2015). The larvae of the stemborer moth burrows in the maize stem as they grow, competing with the plant for the food that is necessary to produce quality grain (Kumela et al., 2019).

According to Khan et al. (2014), FAW and stemborer losses are on average estimated at 37 and 80 percent respectively of annual maize production in Africa under no control technologies. These losses are valued at US$ 4.3 billion annually (Day et al., 2017). Recent studies in Kenya and Ethiopia have estimated losses of 32 and 47 percent respectively of maize production owing to FAW (Kumela et al., 2018). The pest is also estimated to cause losses of 40 and 45 percent of maize production in Zambia and Ghana, respectively (Day et al., 2017). Conversely, maize stemborer pest is estimated to cause a loss of about 44–50 percent of potential maize output in Kenya (Nyukuri et al., 2014; IITA, 2019).

Smallholder farmers in Rwanda and other parts of SSA have used many methods to control FAW and stemborer pest. These measures include handpicking, plant extracts, soil/ash and sawdust/pepper mixture, intercropping, and pesticide application (Midega et al., 2018; Kumela et al., 2018, 2019; Kassie et al., 2020). Pesticides remain by far the most widely used FAW and stemborer control methods. However, the continued use of pesticides has elicited pest resistance and has negative human, animal, and environmental effects (Nicolopoulou-Stamati et al., 2016; Sharma and Singhvi, 2017). Furthermore, the accessibility of pesticides and highly specialized safety equipment for their application remains a challenge to resource-poor farmers in SSA (Day et al., 2017; Kumela et al., 2018).

In cognizance of the negative effects of pesticides and in an attempt to reduce the high maize production losses associated with pests in SSA, the International Center of Insect Physiology and Ecology (*icipe*) and its allies established a habitant management strategy well-known as the push-pull technology (PPT) (Khan et al., 2001). PPT is an integrated pest management method involving the intercrop of cereal crops such as maize with *Desmodium* that “pushes” the pest away from the cereal while *Brachiaria* is planted as a border crop to “pull” the pest (Khan et al., 2008a, 2014; Chepchirchir et al., 2017). The secondary benefits of PPT include improvement of soil fertility through nitrogen fixation, reduced soil erosion and lower use of pesticides, and provision of high-quality fodder for livestock production (Pickett et al., 2014; Kassie et al., 2018; Maina et al., 2020). This biological pest control technology simultaneously reduces the impact of four major production constraints in Africa's cereal-livestock farming system: weeds, pests, poor soil health, and fodder shortage (Chepchirchir et al., 2017).

Introduced in 2017 in Rwanda, the PPT has been widely used in East and Southern Africa to control stemborer pests, Striga weed, and currently FAW pests (Murage et al.,2015a; Midega et al., 2018; Kumela et al., 2018; ICIPE, 2019). Previous studies from SSA reveals that cereal yields and livestock fodder can be twofold or even, in other cases, threefold with use of PPT (Khan et al., 2001, 2008a,b; Cook et al., 2006; Murage et al.,2015a,b). Although the use of PPT technology is labour demanding during initial establishment, the labour requirements decrease substantially after the cropping system is well established (Muriithi et al., 2018). PPT was found to have a benefit-cost ratio of about 2.2:1 relative to 0.8:1 for mono-cropping of maize (Khan et al., 2008a). Yields for maize farmers using the PPT in Uganda and Kenya have been reported to be 1.54 and 2.2 times higher than planting maize without PPT (Khan et al., 2008a; Chepchirchir et al., 2018).

In Rwanda, *icipe,* in collaboration with the Government of Rwanda, introduced a PPT pilot project in 2017 to control FAW and stemborer pests (ICIPE, 2019). The government of Rwanda, through the Rwanda Agricultural Board (RAB) recommended local partners (Food for the Hungry/Rwanda organization) who undertook farmer identification, training and establishment of demonstration plots (ICIPE, 2019; Niassy et al., 2020). Other farmers would later learn from demonstration plots before adopting and receiving necessary support through extension visits from both *icipe* field monitors and government extension officers. However, despite the promotion efforts by *icipe* and the government of Rwanda, the adoption of PPT remains low at only 5 percent (ICIPE, 2019; Niassy et al., 2020). Moreover, the intensity of adoption of the technology in Rwanda remains largely unexplored.

While several recent empirical studies (e.g., Murage et al., 2012, 2015a and b, Muriithi et al., 2018; Chepchirchir et al., 2017; Kassie et al., 2018; Gwada et al., 2019; Niassy et al., 2020) have evaluated the adoption of PPT among maize farmers in SSA, we only find one study (Niassy et al., 2020) from Rwanda. However, a majority of these studies are limited to the analysis of adoption using linear econometric models. This paper contributes to the existing knowledge by evaluating the intensity of adoption of PPT using a fractional response model that is specific to the maize area under PPT in the Nyagatare and Gatsibo districts of Rwanda. The study's answers a fundamental but often ignored research question, “*do the perceived benefits of a technology influence the intensity of its adoption?”* and answers this question in the affirmative for the case of PPT in Rwanda. We find the perceived benefits of PPT, its effectiveness, group membership, livestock ownership, and gender to significantly influence the intensity of adoption of the PPT in Rwanda and recommend awareness creation as a reliable pathway to increasing usage of new technologies. The remainder of this paper is arranged in the following order; Section 2 reviewed past related studies. Sections 3 and 4 presents the study's methods and findings, respectively. Finally, the study draws conclusions and policy recommendations in section 5.

## 2 Past related studies

Most empirical evidence on PPT in Eastern Africa has focused either on gender and adoption (Murage et al., 2015a and b, Muriithi et al., 2018), the effectiveness of its dissemination pathways (Murage et al., 2012), willingness to pay (Niassy et al., 2020) and its welfare benefits (Kassie et al., 2018). Murage et al. (2012) assess the effectiveness of different dissemination pathways in adopting PPT among smallholder maize farmers in Western Kenya using a two-limit Tobit model based on the proportion of land under PPT as a proxy for effectiveness. While the use of the proportion of land under PPT is an appropriate measure of the intensity of adoption, it obscures the intensity of adoption of PPT on maize since it is an aggregate measure for the entire farm that in practice is committed to multiple crop enterprises.

Murage et al. (2015a) applied a multinomial logit model (MNL) to evaluate the determinants of adoption of PPT in Eastern Africa. The MNL model estimates the probability of adoption but is inappropriate in analyzing the intensity of adoption. Murage et al. (2015b) evaluated gender-specific perceptions and the extent of adopting climate-smart PPT in controlling stemborer in Eastern Africa using a Tobit model. The Tobit model is only suitable for analyzing the intensity of adoption when the dependent variable is bounded on one extreme (e.g., land area) but is inappropriate when the dependent variable is bounded on both extremes (e.g., between 0 and 1).

Chepchirchir et al. (2017) evaluated the impact of the adoption of PPT on smallholder maize household's welfare in Eastern Uganda using a generalized propensity score method based on the absolute area allocated to PPT. The use of an absolute area as the dependent variable in estimating the intensity of adoption is inappropriate since such a measure is a proportion that is bounded between 0 and 1 and best measured as a fraction.

Muriithi et al. (2018) examined gender differences in PPT adoption and other sustainable agricultural practices (SAPs) on smallholder maize farmers’ fields in western Kenya using an ordered probit model. This study though insightful, generalizes the estimation to that of the intensity of adoption of SAPs and is not specific to PPT. Kassie et al. (2018) employed a pooled probit model and an economic surplus model to evaluate the probability and welfare impacts of PPT adoption in smallholder maize farms in Kenya. The binary probit model is suitable for assessing the probability of adoption, but does not capture the difference in households regarding allocating land to PPT.

Gwada et al. (2019) assessed the factors influencing the extent of PPT expansion among smallholder resource-poor maize farmers in Homabay County, Kenya, using a censored Tobit model. The farm-wide measure of the intensity of adoption of PPT used in the Gwada et al. (2019) suffers from the same aggregation problems cited under Murage et al. (2012). Niassy et al. (2020) used a binary logit model to evaluate the probability of adoption and the willingness to pay for PPT among smallholder maize farmers in Rwanda. However, while the binary logit model is suitable for analyzing the probability of adoption, it is inappropriate for evaluating the intensity of adoption. While the past evaluations on the adoptions of PPT in Eastern Africa provide useful insights on the drivers of adoption, only a few (Murage et al., 2012; Gwada et al., 2019) attempted to analyze the intensity of adoption using the censored Tobit models that are appropriate when the dependent variable is proportional. However, these two studies used an aggregate measure of the intensity of adoption that obscures the actual intensity of adoption of PPT among maize farmers. The current study employs a fractional logit model based on the proportion of the maize area under PPT to overcome the econometric limitations associated with such estimations.

## 3 Methodology

### 3.1 Conceptual framework

The Use-Diffusion (UD) theory proposed by Shih and Venkatesh (2004) has been widely used to explore farmers’ decision-making process on whether to adopt new technology and how much of that technology to adopt (Hu, 2007; Turner et al., 2010). It is an extension of the adoption-diffusion (AD) theory of Rogers (1995), which examines the process by which an innovation reaches a high number of adopters, the diffusion is expedited, and the innovation is considered successful (Mahajan et al., 1990: Rogers, 1995). The UD theory addresses the limitation of AD theory that fails to account for the diffusion process with discontinued behavior (Golder and Tellis, 1998; Turner et al., 2010). It provides an understanding of both the rate of use (high/low) and the variety (intensity) of using a technology. It can be used to model the determinants of technology adoption and the outcomes of technology adoption.

**Figure 1:**
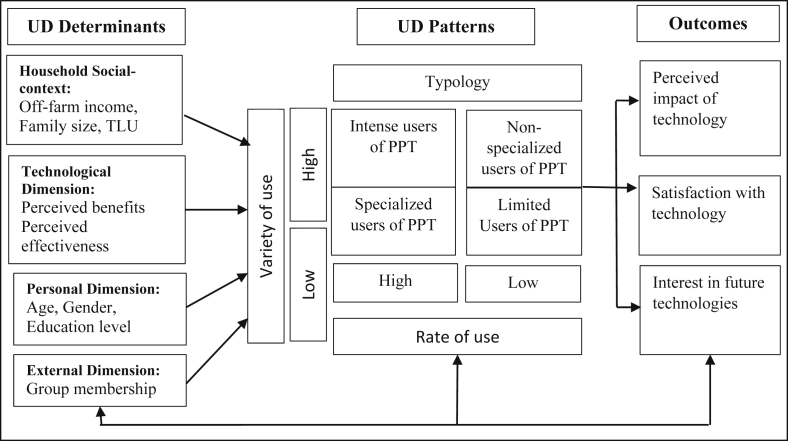
Use-diffusion model adapted from Shih and Venkatesh (2004).

The theoretical bais of the AD model comprise an S-shaped diffusion curve that integrates the speed of penetration and a critical number of users in a two-step model of diffusion (Theotokis and Doukidis, 2009). The corresponding theoretical components of the UD model are the progressing nature of use (variety and rate), sustained uninterrupted use (disadoption), and technology outcomes (perceived usefulness and integration) (Shih and Venkatesh, 2004). While the variable of interest in the AD model is the rate or time of adoption, the return variable in the UD model is the rate of use and variety of use. Shih and Venkatesh (2004) propose the use of two distinct measures (variety of use and rate of use) to estimate the degree (intensity) of use of new technology. The rate of use indicates the time a person spends using the product during a designated period. Variety of use signifies the different ways the product is used (Shih and Venkatesh, 2004). Figure 1 presents the conceptual framework of the UD theory on which this study is based.

The UD model comprise of threefold important elements: (1) factors of UD model, (2) patterns of UD, and (3) outcomes of UD model. Factors that affect variety and rate of usage constitute UD factors (household social-context, personal aspect, technological aspect, and external aspect). Combining rate and variety of usage (high/low) produces a four-way typology of usage (specialized usage, intense usage, non-specialized usage, and limited usage), henceforth UD patterns (Figure 1). Intense usage deal with the individuals who apply product of innovation to an important degree in relation to both rate of usage and variety of usage. Non-specialized usage deal with the individuals who apply numerous roles of the product, however, take little time in using the product. Finally, limited usage deal with the individuals who apply the product of innovation to a minor degree in relation to both rate of usage and variety of usage; specifically, users discovery small, if any, worthy potential application and hence commit the product to a quite negligible function, even to the level of "disadoption" (Lindolf, 1992).

Different types of users have different experiences of the UD outcomes of the technology (perceived impact, degree of satisfaction, interest in future features of the technology etc.). In practice, the determinants of technology adoption are modeled using discrete choice (logit, probit models and their extensions), Tobit models, censored regressions, truncated regressions, and discriminant analysis techniques (Maddala, 1991; Noreen, 1998; Wooldridge, 2002, 2012). The binary choice models (Probit and Logit models) approximate the probability of event's incidence and are suitable for binary response-dependent variables (Greene, 2003).

Several empirical models have been used to analyze the intensity of adoption of new technologies, including Poisson (Awuni et al., 2018; Mahama et al., 2020; Kolady et al., 2020), Tobit (Murage et al., 2015b; Gwada et al., 2019) and fractional response (Papke and Wooldridge, 1996, 2008; Ramalho et al., 2011; Pokhrel et al., 2018; Ogoudedji et al., 2019; Nyabaro et al., 2019). The choice of model to use depends on the nature of the dependent variable. Continuous variables that are restricted (proportions) in nature are normally estimated using truncated regressions, censored models, or Tobit regressions (Papke and Wooldridge, 1996, 2008; Gallani et al., 2015).

These methodologies, though, suffers from some constraints, specifically wherever the dispersion of the variable is restricted both below and above and a quantifiable share of the sample observations falls at one of the borders. The Fractional Response Models (FRM) offers a feasible option to addressing several of the econometric restrictions associated with nonlinear methods presently used in modelling continuous bounded dependent variables (Papke and Wooldridge, 1996). The FRM yields estimates of higher fit compared to other linear approximation models through control of dependent variable that is bounded from both below and above, predicting response values that fall inside the interval bounds of the dependent variable while capturing the nonlinearity of the data. Additionally, the fractional response model allows a direct approximation of the conditional expectation of the dependent variable given the predictors and thus does not need special data transformations at the corners.

The FRM accounts for limitations of the existing methodologies for the statistical analysis of bounded dependent variables and provides a material number of corner observations. Given its simplicity in computation, FRM provides high levels of flexibility in its application to longitudinal, panel, and cross sectional data. Further, the FRM accounts for nonlinearity, and relaxes the numerous restricting assumptions that are necessary in traditional econometric results. The FRM extends the general linear models (GLM) to a class of functional forms that overcome the limitations of the outdated econometric models for variables that are bounded in nature (Wooldridge, 2012).

The approximation of the parameters in the model is grounded on a quasi-maximum likelihood method (QMLE), generating estimates that are proportionately efficient and entirely robust under the GLM circumstances (Papke and Wooldridge, 1996). According to Gillani and Krishnana (2016), the FRM offers a better fit whereas controlling for the non-constant returns of the dependent variable along the range of the predictors and the nonlinearity in the data. Moreover, the estimation of average partial effects at different levels of the independent variables indicate that the FRM affirms more precise inferences, specifically in circumstances where observations at the end of the distribution are of specific interest for the researcher. Given the incremental explanatory power and the simplicity in computation, the use of the FRM should be contemplated at least as an option to other traditional econometric approaches applied in survey-based research.

### 3.2 Empirical framework

We employ a fractional logit model (FLM) to evaluate the intensity of adoption of PPT among smallholder maize farmers in Rwanda. The intensity of adoption of PPT is defined as the number of acres of maize under PPT per household divided by the total maize acreage per household, which is bounded between 0 and 1. Following Papke and Wooldridge (1996), we specify the following functional form for the expectation of the intensity of adoption of PPT $Y_{i}$ of the *i*th household conditional on $\;X_{i}$ (a vector of independent variables);

(1) $$E\left(Y|X_{i}\right)=Z\left(\beta X_{i}\right)$$

Where *Y*_*i*_ denotes the intensity of adoption of PPT, *X*_*i*_ is a vector of farm, farmers and technology-specific attributes (Table 1) and β a vector of unknown parameters to be approximated. *Z(·)* is a cumulative distribution function that follows a logistic distribution function representing a nonlinear link function satisfying 0≤Z(⋅)≤1, ensuring that the approximated values lie in the interval of 0 and 1 and *E* is the expectations operator.

**Table 1:** Description of variables used in the Fractional Logit model.

Variable	Description	Unit of measurement
**Dependent variable**		

Intensity of adoption of PPT (Proportion)	Acres of maize under PPT divided by the total acreage under maize per farm	Proportion

**Independent variables**		

Perceived PPT Benefits	Farmers perceptions on PPT's ability to increase maize yields	Dummy (1 = Yes, 0 otherwise)
Perceived PPT Effectiveness	Farmers perceptions' on the effectiveness of PPT to control FAW and stemborer	Dummy (1 = Effective, 0 otherwise)
Age	Age of the household head in years	Years
Gender	Gender of the household head	Dummy (1 = Male, 0 otherwise
Education	Number of years spent in school	Continuous
Family size	Number of person in the household	Continuous
Off-farm income	Participation in off-farm income activity	Dummy (1 = Yes, 0 otherwise)
Group membership	Membership to farmer groups	Dummy (1 = Member, 0 otherwise)
Livestock ownership (TLU)	Livestock ownership	Continuous

Note: TLU is tropical livestock unit. TLU corresponding for different livestock were computed as camels = 1, cattle = 1, donkeys = 0.8, goats and sheep = 0.2 and poultry = 0.04 (WISP, 2010).

Eq. (1) is approximated using a quasi-maximum likelihood estimation technique where the likelihood for an observation is specified as the Bernoulli likelihood;

(2) $$Li={\left[F\left(\beta X_{i}\right)\right]}^{Yi}\;{\left[1-\;F\left(\beta X_{i}\right)\right]}^{1-\;Yi}$$

The QMLEs of β are consistent provided that the conditional expectation in Eq. (1) is correctly stated even if the Bernoulli specification is incorrect (Papke and Wooldridge, 2008). A FLM constructed on the logistic conditional mean function and quasi-likelihood method is advantageous (Murteira and Ramalho, 2016). Following (Hausman and Leonard, 1997), the asymptotic variance-covariance of the matrix of the QMLE estimates is approximated, with maintenance of only first momentum assumptions without any additional second momentum assumptions.

### 3.3 Data sources and sampling technique

We use survey data collected in 2018 from a sample of 194 PPT adopter households in the Nyagatare and Gatsibo districts in Rwanda. A cluster sampling procedure was used to select the respondents. In the first-stage, the two districts (Gatsibo and Nyagatare) were purposively selected since they were the pilot areas for the PPT project. Within each district, the pilot had been conducted in one sector, and thus, Gatunda and Nyagihanga sectors, from the Nyagatare and Gatsibo districts were selected, respectively. A simple random sampling technique was used in the second-stage to select 240 adopter households from a sampling frame of households who had participated in the pilot provided by the Rwanda Agricultural Board in the two districts. However, 46 of the selected households had stopped using the technology in the preceding 12 months and were therefore dropped from the sample resorting to a sample size of 194 adopters farming households who were interviewed with a pre-tested semi-structured questionnaire. The 194 adopter households selected comprised of 133 and 61 farmers from the Gatsibo and Nyagatare districts respectively, were then interviewed with a pre-tested semi-structured questionnaire. The data was then analyzed with Stata version 14.

### 3.4 Measurement of variables

Table 1 presents the description and measurement of the variables used in the analysis. The dependent variable of the FLM used in this study is the intensity of PPT adoption among smallholder maize farmers in Rwanda. It is derived by dividing the number of acres of maize under PPT per household by the total maize acreage owned by a household. It is a fractional variable bounded between 0 and 1. To derive the proportion of land area under PPT, farmers were asked two successive questions: i) *how many acres of land have you set aside for maize production?* The second question asked was: ii) *of the total acreage you have set aside for maize production, how many acres have you allocated to push-pull technology (acres)?*

The use-diffusion theory and previous studies (e.g., Kassie et al., 2011; Murage et al., 2012, 2015b; Ghimire et al., 2015; Obuobisa-darko, 2015; Chepchirchir et al., 2017; Maina et al., 2020; Kolady et al., 2020) inform the choice of the independent variables (Table 1) used in the analysis. They included the perceived benefits of PPT use, the perceived effectiveness of PPT use as compared to other pest control methods, group membership, off-farm income sources, education level, gender, total livestock units (TLU), age and family size.

Farmer's assessment of maize yields with PPT adoption as compared to the yields before PPT adoption was used to proxy for the perceived PPT benefits. Farmers were asked to compare their maize yields before and after the adoption of PPT. Their responses were grouped into a dummy outcome variable equivalent to 1 if household's perceive PPT increased yields and zero otherwise. Positive relationships have been reported between the perceived benefits of new technologies and their adoption (Ghimire et al., 2015; Kolady et al., 2020). Similarly, PPT's perceived effectiveness control stemborer and FAW relative to other methods was also measured as a binary response variable, equal to one 1 if PPT was effective and zero otherwise.

Group membership was used as a representation for sources of information sharing on PPT, procuring inputs, and marketing output. It was measured as a dummy variable equivalent to one if a farmer was a member of an agricultural group and zero otherwise. Social networks such as groups or farmer associations facilitated the exchange and gathering of information related to PPT and provided platforms through which farmers accessed inputs and marketed output. Previous studies have revealed positive associations between group membership and PPT adoption (Kassie et al., 2011; Ghimire et al., 2015; Obuobisa-darko, 2015; Chepchirchir et al., 2017).

Off-farm income played a key role in providing financial resources necessary for investment in PPT and was measured as a dummy variable equivalent to one if a farmer had an off-farm income source and zero otherwise. Gender, education, age, livestock ownership, and family size were also used as control variables following preceding studies (see Kassie et al., 2011; Murage et al., 2012, 2015b; Maina et al., 2020).

Gender was measured as a dummy variable equivalent to 1 if the household head was male and zero otherwise. The available literature on the influence of gender on the PPT adoption is mixed. Age was used as a proxy of farmers’ experience. Education was measured by the number of schooling years spent by the respondent, while age was measured in years. Several previous studies have shown positive relationships between age and education on one side and the adoption of PPT on the other (Obuobisa-darko, 2015; Mahama et al., 2020). The number of livestock owned was used as a proxy for wealth status and measured in TLU. Family size was also incorporated as a proxy for available family labour and measured as the entire number of persons per household.

## 4 Results and discussions

### 4.1 Descriptive results

Table 2 presents the demographic characteristics of PPT adopter maize farmers in Rwanda. Average farm sizes for PPT adopters were 3 acres, which was slightly higher than the national average at 2.6 acres (MINAGRI, 2018). The adopter households' allocated approximately 1.035 acres (35 percent) of their farms to maize production, out of which 0.269 acres was under PPT to yield an intensity of adoption of 0.26. On average, the PPT adopter farmers were middle aged (50 years) with about 6.42 years of schooling corresponding to the attainment of a primary school level of education. The average family size in the study area was 5.24 persons, which compares favourably with the national average at 4. Three quarters of the respondents were male, which was expected given the patriarchal nature of the society. Moreover, 61 percent of the households belonged to farmers’ groups through which they share information on PPT, procured inputs and marketed output.

**Table 2:** Demographic characteristics of PPT adopter maize farmers in Rwanda.

Variables	Mean (n = 194)	SD	Minimum	Maximum
Farm size (Acres)	3.115	3.252	0.250	24.700
Land area under maize (Acres)	1.035	1.059	0.100	5.100
Maize area under PPT (Acres)	0.269	0.279	0.050	1.250
Age (Years)	50.02	10.76	24	85
Education (Years)	6.42	2.97	0	18
TLU (Number)	1.91	4.09	0	39
Family size (Number)	5.24	2.04	2	13
**Frequencies**	**Count**	**Percent**		
Gender of household head (% Male)	146	74.74		
Off-farm income source (% accessing)	91	46.91		
Group membership (% belonging)	118	60.82		
Perceived PPT benefits (% positive)	112	57.73		
PPT effectiveness in stem borer control (%)	117	60.31		
PPT effectiveness in FAW control (%)	115	59.28		

Almost all households in the study area owned livestock with an average TLU of 1.91, which is understandable given the small land holding sizes. As expected with adopters of any technology, maize farmers in Rwanda had a positive perception of PPT use. Fifty-eight percent of the respondents perceived PPT use to increases maize yields, while 60 percent of the farmers perceived the technology to be effective in the control of FAW and stemborer relative to other methods. Moreover, 47 percent of the respondents undertook other off-farm income earning undertakings, which was used to complement farm incomes required to cover the initial labour costs for setting up the PPT plots that can be a hindrance to adoption.

### 4.2 Econometric results

Table 3 presents the quasi-maximum likelihood estimates (QMLE) of the intensity of adoption of PPT from the fractional logit model. The mean Variance Inflation Factor (VIF) score was 1.12 (critical value 10) while the partial correlation coefficients for all the independent variables were less than 0.5 suggesting that multi-collinearity of the explanatory variables was not problematic (Kennedy, 1985; O'brien, 2007). The Breusch-pagan test fails to rejects the null hypothesis of homoscedasticity (Chi^2^ (1) = 0.01; Prob > chi^2^ = 0.907) ruling out the presence of heteroscedasticity. The Wald statistic was significantly at the one percent level signifying a high prediction power of the model (Mwololo et al., 2019). Finally, the Pearson and deviance tests for unequal dispersion were not significant (p > 0.05) suggesting that the FLM fitted the data well. Results show that the perceived PPT benefits and effectiveness, group membership, livestock ownership, and gender had positive significant effects on the intensity of adoption of PPT in Rwanda. Gender, education, age, livestock ownership, and family size were also used as control variables but were insignificant in explaining the intensity of adoption of PPT in Rwanda. The quasi-maximum likelihood estimates of the FLM are consistent as long as the conditional expectation of the intensity of adoption of PPT is correctly specified even if the Bernoulli specification is inappropriate. Thus they are more reliable than recent estimates of the intensity of adoption of agricultural innovations using both linear and non-linear models of estimation.

**Table 3:** Fractional Logit QMLE of the intensity of adoption of PPT in Rwanda.

Variable	Coefficient	Robust Std deviation	Marginal effects	Robust Std error
Perceived PPT benefits	0.292∗∗∗	0.113	0.0632∗∗∗	0.024
Perceived effectiveness of PPT	0.301∗∗∗	0.112	0.0648∗∗∗	0.024
Age	-0.004	0.005	-0.001	0.001
Gender	0.274∗∗	0.132	0.058∗∗	0.027
Education	0.018	0.018	0.004	0.004
Family size	-0.024	0.023	-0.005	0.005
Off-farm income	0.037	0.105	0.008	0.023
Group membership	0.246∗∗	0.113	0.053∗∗	0.024
Livestock ownership (TLU)	0.074∗∗	0.033	0.016∗∗	0.007
Constant	-1.364∗∗∗	0.341		
Number of observations	194			
Wald Chi^2^ (9)	58.630	0.000		
Pseudo R^2^	0.021			
Log pseudo likelihood	-120.012			
Breusch-pagan/Cook-Weisberg	Chi^2^ (1) = 0.011 Prob > Chi^2^ = 0.907			
Mean Variance inflation factor	1.120			
Deviance	4.468			
Pearson	4.468			

∗, ∗∗ and∗∗∗ denotes 10%, 5% and 1% significance level respectively.

Farmer's perceptions of the ability of PPT use to increase maize yields had positive significant effects on the intensity of adoption and was significant at the 1 percent level signifying that the intensity of adoption increased with a respondents' positive perception of the benefits of the technology. Farmers who perceived PPT use to increase yields had higher intensities of adoption of the technology of 6.32 percent than their counterparts who thought otherwise. This result is in agreement with the finding of previous studies (Meijer et al., 2014; Ghimire et al., 2015; Murage et al., 2015a, b and Kolady et al., 2020). Meijer et al. (2014), which reported that farmer's initial information of the benefits of the technology increased the probability of adoption. Ghimire et al. (2015) reported that positive perception of technology's superiority in yield increased intensity of adoption of improved maize technologies.

In conformity with the expectations, the perceived effectiveness of PPT in control of FAW had a significant positive influence on the intensity of adoption of PPT at the 1 percent level. Positive perceptions on the effectiveness of PPT increased the intensity of adoption by 6.48 percent. Maize farmers would readily adopt a technology that effectively controls pests compared to the alternative pesticides that have been linked with the negative environmental effects. This finding supports the results of Gwada et al. (2019), who reported a positive association between farmers' perceptions on the severity of stemborer infestation and the intensity of adoption of PPT in Kenya. Similar result were reported by Kolady et al. (2020) in South Dakota, who observed that positive perceptions of profitability increased producers’ intensity of adoption of precision agriculture technologies.

Group membership had a positive and significant influence on the intensity of adoption of PPT at the 5 percent level. Belonging to a farmers group increased the intensity of adoption of PPT by 5.8 percent. Groups play an important role in transferring information and knowledge and availing inputs (Okello et al., 2021). Often, smallholder farmers procure inputs, market outputs and acquire information through farmers' groups that are used to leverage the benefits of economies of scale. Obuobisa-Darko (2015) find a positive association between group membership and intensity of adoption of cocoa research innovations in Ghana. A similar result was reported by Ghimire et al. (2015) maize farmers belonging to groups and local cooperatives in Nepal were exposed to numerous information sources, enabling the farmers to evaluate the risks, benefits and take advantage of new agricultural innovations. Group membership acts as a proxy for social capital and farmer-based extension support methods such as field days, farmer-teacher, and farmer-farmer are modelled on group learning methods where social capital forms the basis for interactions and information exchange among members and other extension agencies (Wossen et al., 2017). Thus, social capital not only provided social networks but also facilitated in information flow and provided opportunities for peer learning where farmers shared experiences and information about PPT adoption. This means that the dissemination of PPT reached more farmers when conducted through farmer groups and was more likely to increase intensity of adoption (Kassie et al., 2011; Chepchirchir et al., 2017).

Livestock ownership had a positive significant influence on the intensity of adoption of PPT at the 5 percent level. A positive relationship between livestock ownership and the intensity of adoption is expected given that Napier grass and desmodium *spp* are used as animal feeds, and thus the technology compliments livestock production. Livestock ownership is indicative of a farmer's wealth status, an important component of technology adoption. A one percent increase in the TLU increased the intensity of adoption of PPT in Rwanda by 1.6 percent. A similar result was reported in Maina et al. (2020) who observed that farmers with a higher number of livestock assets were more willing to adopt and increase land acreage under brachiaria grass and desmodium that are components of PPT. Other studies noted a direct association between livestock ownership and adoption of agricultural technologies due to the utilization of fodder and crop residues as animal feeds (Khan et al., 2014; Murage et al., 2015a; Kassie et al., 2018).

Male farmers in Rwanda committed larger maize acreage to PPT relative to their female counterparts. The significant positive association between gender and the intensity of adoption of PPT is expected given the limited access of female-headed households to productive resources such as land, credit and extension. Male headed households had 5.8 percent more likelihood of allocating their maize plots to PPT relative to their female counterparts. This can be due to number of socio-cultural factors (Mbugua et al., 2020). This finding is in agreement with the result by Murage et al. (2015b), who observed a positive correlation between gender and intensity of adoption of climate-smart PPT in Kenya.

## 5 Conclusion and policy implications

This study evaluates the factors influencing the intensity of adoption of PPT among smallholder maize farmers in Gatsibo and Nyagatare districts of Rwanda. Survey data from 194 PPT adopter maize farmers was analyzed using a fractional logit model. Overall, fifty-eight percent of the respondent's perceived PPT use to increase yields, while 60 percent of them perceived PPT to be effective in the control of both FAW and stemborer relative to other methods. Our results revealed that the perceived PPT benefits, the perceived effectiveness of PPT in control of FAW, group membership, livestock ownership and gender positively and significantly influenced the intensity of adoption of PPT among smallholder maize farmers in Rwanda. We conclude that farmer's perception of technology attributes and sources of information play key roles in technology adoption decisions.

Given these findings, development initiatives in Rwanda should focus on strategies that create and disseminate information that enhances farmer awareness on the perceived benefits of the technology and its effectiveness in pest control relative to other existing methods such as pesticides. Such strategies could include the use of extension methods (e.g. farmer field schools, demonstrations etc.) that disseminate information on PPT and focus on farmer groups especially those whose members own livestock. Furthermore, efforts to disseminate PPT information should target male farmers differently from female farmers given their different access to productive resources that are important drivers of technology adoption.

Lastly, while the biological and societal background has been eloquently discussed, a direct comparison against other (or similar) models has not been elaborated. Moreover, despite the rigorous econometric methods validating the results on intensity of PPT adoption, the authors recognize the limitations in approximation. First, the study used cross-section data that does not capture the dynamics changes in integrated pest management used by smallholder maize farmers. Secondly, the study's limitation pertains to small sample size conducted when the technology was being disseminated among smallholder farmers. Thirdly, although our estimates demonstrate the factors influencing intensity of PPT adoption, the study did not take into account plot-varying characteristics and institution factors such as credit and extension accessibility. In view of overcoming this weakness, the study recommends future studies to include additional variables and years of sampling to validate the study's findings and get results that are more robust.

## Declarations

### Author contribution statement

Vincent Gadamba Misango: Conceived and designed the experiments; Performed the experiments; Analyzed and interpreted the data; Contributed reagents, materials, analysis tools or data; Wrote the paper.

Jonathan Makau Nzuma; Patrick Irungu; Menale Kassie: Conceived and designed the experiments; Analyzed and interpreted the data; Wrote the paper.

### Funding statement

This work was supported by the European Commission (Grant No. DCIFOOD/2018/402-634). the International Centre of Insect Physiology and Ecology (icipe), the Swedish International Development Cooperation Agency (Sida), the Swiss Agency for Development and Cooperation (SDC), Germany’s Federal Ministry for Economic Cooperation and Development (BMZ), the Federal Democratic Republic of Ethiopia and the Kenyan Government. Vincent Gadamba Misango was supported by the African Economic Research Consortium (AERC).

### Data availability statement

Data will be made available on request.

### Declaration of interests statement

The authors declare no conflict of interest.

### Additional information

No additional information is available for this paper.
